# Supplementary material for: TRIM5α recruits HDAC1 to p50 and Sp1 and promotes H3K9 deacetylation at the HIV-1 LTR
Source: Nat Commun. 2023 Jun 8;14:3343. doi: 10.1038/s41467-023-39056-6 (PMC10250300; doi:10.1038/s41467-023-39056-6)
Supplement: Supplementary file 1 — Supplementary Information [file 41467_2023_39056_MOESM1_ESM.pdf]

Supplementary Information

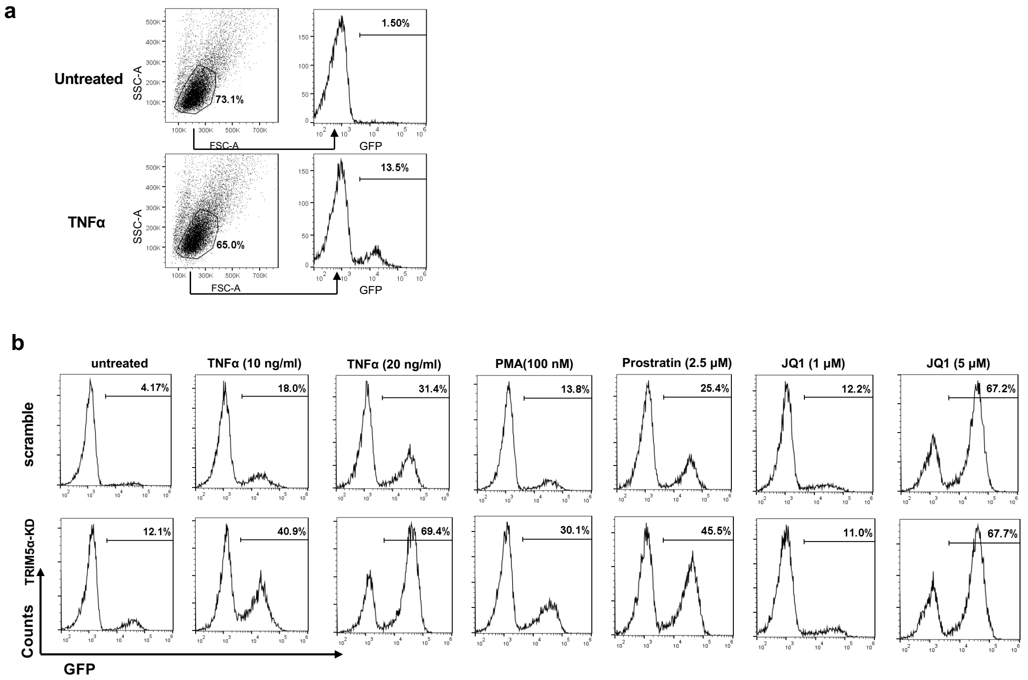

Supplementary Fig. 1. TRIM5α prevents HIV-1 gene expression under basal conditions or in the presence of selected LRAs. a Gating strategy for detection of LRA-induced GFP expression within live cell populations. **b** TRIM5α-KD or scramble-KD J-Lat cells were treated with TNFα (10 ng/ml; 20 ng/ml), PMA (100 nM), prostratin (2.5 μM), or JQ1 (1 μM; 5 μM). GFP expression was assessed via flow cytometry in 24 hrs. Source data are provided as a Source Data file.

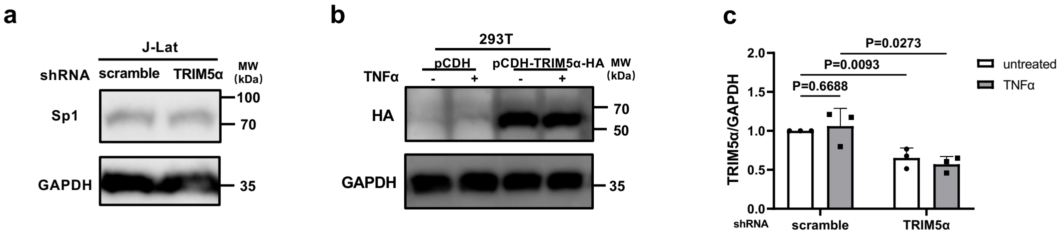

Supplementary Fig. 2. TRIM5α has no impact on the expression of Sp1 and remains unaffected upon TNFα treatment. a Protein extracted from J-Lat cells with stable TRIM5α-KD or scramble-KD was subjected to western blot for the detection of Sp1 by a specific antibody. GAPDH was used as a loading control. **b** Stably TRIM5α-HA transduced 293T cells were treated with or without 20 ng/ml TNFα for 24 hrs. The protein level of exogenously expressed TRIM5α-HA was detected by western blot, with GAPDH as a loading control. **c** RNA was extracted from TRIM5α-KD or scramble-KD J-Lat cells that were treated with or without 20 ng/ml TNFα treatment for 24 hrs. qPCR was then employed to qualify the expression of endogenous TRIM5α. The relative expression was normalized by GAPDH. Data are representative of 2 independent experiments and are mean ± SD (n=3). The statistical significance analyses were performed using a two-sided unpaired t test. Source data are provided as a Source Data file.

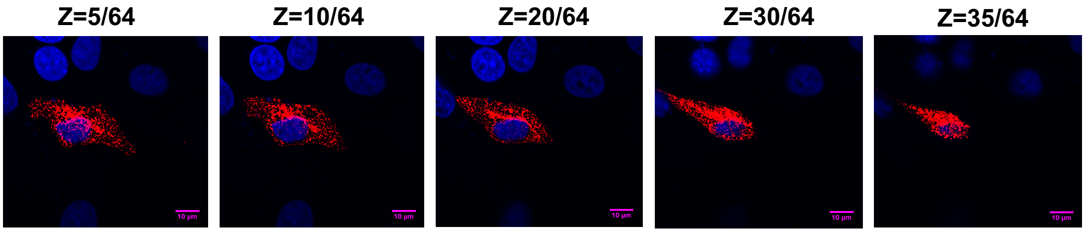

Supplementary Fig. 3. Detection of the localization of TRIM5α. Immuno-fluorescence and confocal analysis were performed to visualize the localization of HA-tagged human TRIM5α overexpressed in 293T cells. Cells are transfected with pLPCX-huTRIM5α-HA for 48 hrs before staining with HA-specific antibodies and DAPI. A total of 64 Z-stack images were acquired and representative Z-stack images were shown. Data are representative of at least 3 independent experiments. Source data are provided as a Source Data file.

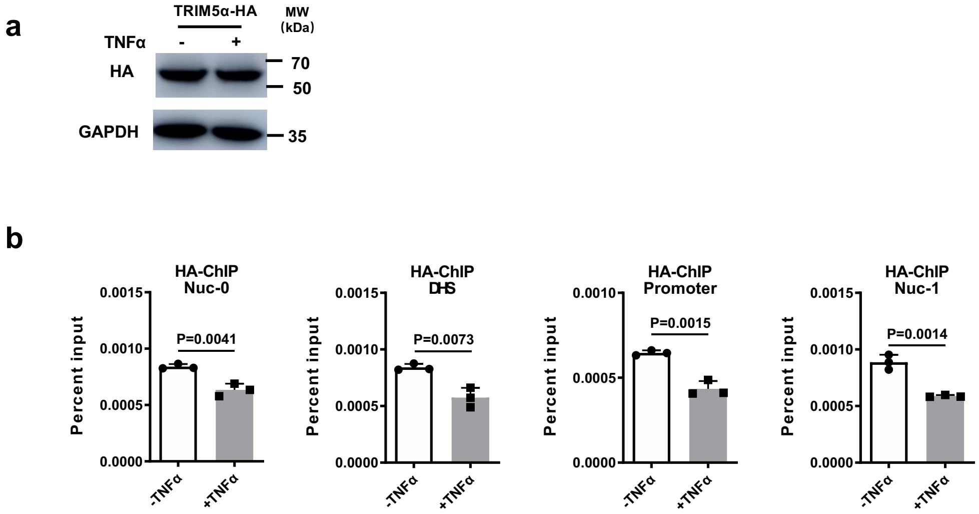

Supplementary Fig. 4. Dissociation of TRIM5α-HA from HIV-1 full LTR reporter construct upon TNFα treatment. a pLPCX-TRIM5α-HA was cotransfected with HIV-1 full LTR reporter in 293T cells. 48 hrs posttransfection, cells were left treated or untreated with TNFα (20 ng/ml). Western blot was performed with anti-HA antibody for the pLPCX-TRIM5α-HA overexpression. **b** ChIP-qPCR analyses were performed to detect the enrichment of HA-tagged TRIM5α on the HIV-1 full LTR. Data are representative of 2 independent experiments and show mean ± SD (error bars). Source data are provided as a Source Data file.

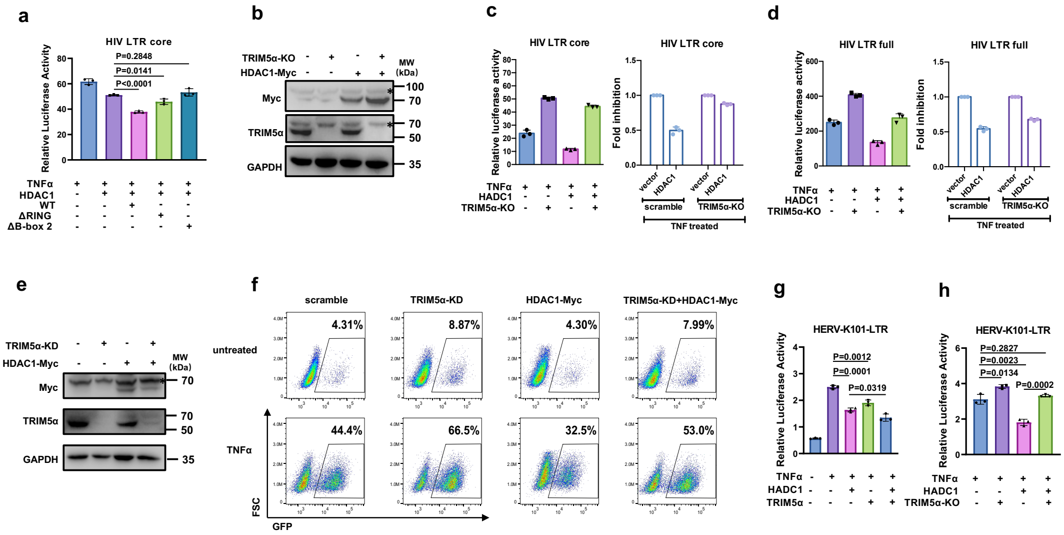

Supplementary Fig. 5. TRIM5α depletion does not fully abolish the effect of HDAC1 on the repression of HIV-1 transcription. **a** Vector or TRIM5α mutant (100 ng) and HDAC1 (100 ng) were cotransfected with pGL-HIV-LTR-core-luc (100 ng) along with pRL-TK. 24 hrs post-transfection, TNFα was added and luciferase activity was measured at 24 hrs post-stimulation. Data are representative of at least two independent experiments. **b** Immunoblot analysis of transient overexpression of HDAC1 in 293T or TRIM5α-KO 293T cells. Asterisk (*) indicates the non-specific bands. **c**, **d** 293T or TRIM5α-KO 293T cells were cotransfected with HDAC1-Myc (100 ng) and pGL-HIV-LTR-core-luc (100 ng) (**c**) or pGL-HIV-LTR-full-luc (100 ng) (**d**). 24 hrs post-transfection, cells were treated with TNFα for 24 hrs before luciferase activity was measured. Fold inhibition were calculated to compare the suppressive effect of HDAC1 on LTR activity between control cells and TRIM5α-KO cells. Data are representative of at least two independent experiments and are mean ± SD (n=3). **e**, **f** shRNA-scramble or shRNA-TRIM5α J-Lat cells were transfected with HDAC1-Myc. Cells were

then stimulated with or without TNFα for 24 hrs. Immunoblot analysis of transient overexpression of HDAC1 in 293T or TRIM5α-KO 293T cells (**e**). Asterisk (*) indicates the non-specific bands. GFP expression in each group was examined to compare the HIV-1 latency reversal (**f**). Data are representative of at least two independent experiments. **g** HDAC1-Myc (100 ng) or TRIM5α-HA (100ng) were cotransfected with HERV-K101-LTR-luc and pRL-TK. Cells were treated with or without TNFα for 24 hrs before luciferase activity was measured. **h** HERV-K101-LTR-luc (100ng) was cotransfected with empty vector (100ng) or HDAC1 (100ng) and RL-TK in 293T or 293T TRIM5α-KO cells. 24 hrs post-transfection, cells were treated with TNFα for another 24 hrs, followed by luciferase activity measurement. Data are representative of three independent experiments and are mean ± SD (n=3). The statistical significance analyses were performed using a two-sided unpaired t test. Source data are provided as a Source Data file.

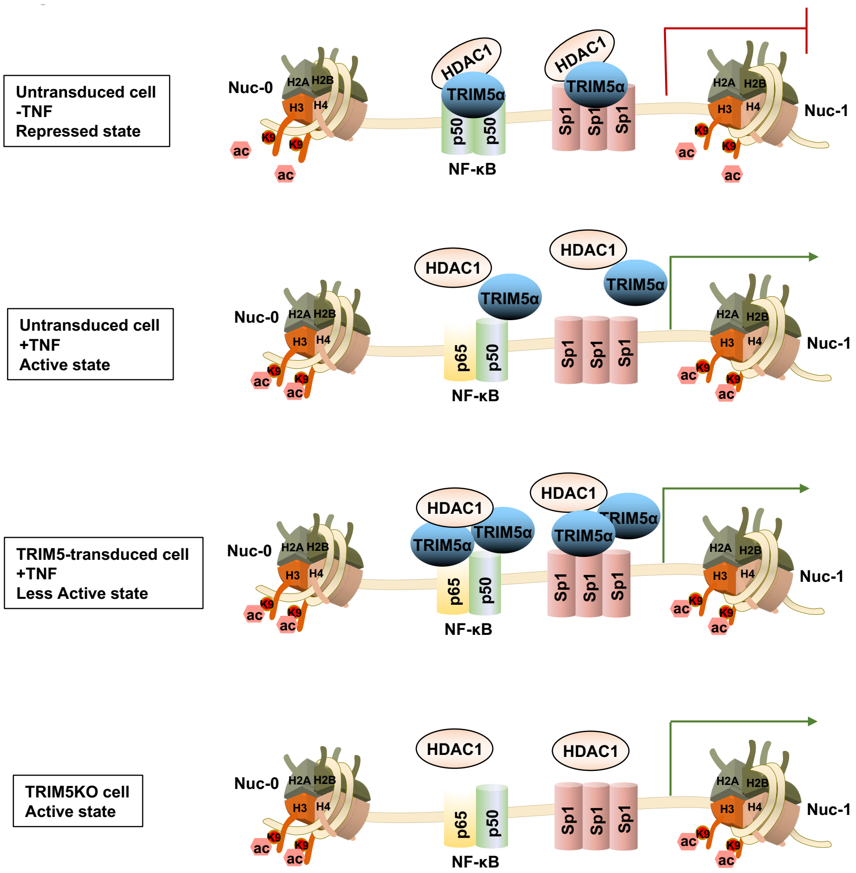

Supplementary Fig. 6. Model for regulation of HIV latency by TRIM5α. At the HIV-1 latent phase, TRIM5α occupies at the HIV-1 LTR region and recruits HDAC1 to transcription factors p50 and Sp1, which leads to H3K9 deacetylation. When TNFα is present, TRIM5α disassociates from HIV-1 LTR, leading to an active state of HIV-1 transcription. Overexpression of TRIM5α rescues part of the TRIM5α recruitment on LTR, resulting in a less active state of HIV-1 transcription. On the other hand, deletion of TRIM5α leads to decreased HDAC1 recruitment and H3K9 deacetylation, which promotes the activation of HIV-1 gene transcription.
