# Supplementary material for: TRIM5α recruits HDAC1 to p50 and Sp1 and promotes H3K9 deacetylation at the HIV-1 LTR
Source: Nat Commun. 2023 Jun 8;14:3343. doi: 10.1038/s41467-023-39056-6 (PMC10250300; doi:10.1038/s41467-023-39056-6)
Supplement: Supplementary file 3 — Reporting Summary [file 41467_2023_39056_MOESM3_ESM.pdf]

Corresponding author(s): Dan Mu
Last updated by author(s): May 16, 2023

Reporting Summary

Nature Portfolio wishes to improve the reproducibility of the work that we publish. This form provides structure for consistency and transparency in reporting. For further information on Nature Portfolio policies, see our Editorial Policies and the Editorial Policy Checklist.

Statistics

For all statistical analyses, confirm that the following items are present in the figure legend, table legend, main text, or Methods section.

n/a	Confirmed	
☐	☒	The exact sample size (*n*) for each experimental group/condition, given as a discrete number and unit of measurement
☐	☒	A statement on whether measurements were taken from distinct samples or whether the same sample was measured repeatedly
☐	☒	The statistical test(s) used AND whether they are one- or two-sided<br>*Only common tests should be described solely by name; describe more complex techniques in the Methods section.*
☒	☐	A description of all covariates tested
☒	☐	A description of any assumptions or corrections, such as tests of normality and adjustment for multiple comparisons
☐	☒	A full description of the statistical parameters including central tendency (e.g. means) or other basic estimates (e.g. regression coefficient) AND variation (e.g. standard deviation) or associated estimates of uncertainty (e.g. confidence intervals)
☐	☒	For null hypothesis testing, the test statistic (e.g. *F*, *t*, *r*) with confidence intervals, effect sizes, degrees of freedom and *P* value noted<br>*Give P values as exact values whenever suitable.*
☒	☐	For Bayesian analysis, information on the choice of priors and Markov chain Monte Carlo settings
☒	☐	For hierarchical and complex designs, identification of the appropriate level for tests and full reporting of outcomes
☒	☐	Estimates of effect sizes (e.g. Cohen's *d*, Pearson's *r*), indicating how they were calculated

Our web collection on statistics for biologists contains articles on many of the points above.

Software and code

Policy information about availability of computer code

Data collection	Andor2000 microscope was used to obtain confocal images.<br>Bio-Rad CFX96 or ABI QuantStudio real-time PCR system was used to collect quantitative PCR data.<br>BD FACSverse cytometer driven by FACSuite 1.0.3 was used to collect folw cytometry data.<br>ChemiDoc Touch Imaging System was used to collect western blot data.<br>Thermo Scientific TM Varioskan TM LUX was used to collect luciferase data.
Data analysis	FlowJo7.6.1 was utilized to analyze flow cytometry data.<br>Fiji (a distribution of ImageJ) software was used to analyze imaging data.<br>GraphPad Prism 6 was used for all of graphical analysis.

For manuscripts utilizing custom algorithms or software that are central to the research but not yet described in published literature, software must be made available to editors and reviewers. We strongly encourage code deposition in a community repository (e.g. GitHub). See the Nature Portfolio guidelines for submitting code & software for further information.

Data

Policy information about availability of data

All manuscripts must include a data availability statement. This statement should provide the following information, where applicable:

- Accession codes, unique identifiers, or web links for publicly available datasets
- A description of any restrictions on data availability
- For clinical datasets or third party data, please ensure that the statement adheres to our policy

All raw and processed data will be made available upon request.

Human research participants

Policy information about studies involving human research participants and Sex and Gender in Research.

Reporting on sex and gender	No sex- or gender-based designs or analyses were performed.
Population characteristics	Anonymous healthy blood donors (age 20-50) for primary CD4 + T cells isolation.
Recruitment	Healthy blood donors.
Ethics oversight	Experiments involving human blood and CD4+ T cells were reviewed and approved by the Ethics Committee of Chongqing Medical University.

Note that full information on the approval of the study protocol must also be provided in the manuscript.

Field-specific reporting

Please select the one below that is the best fit for your research. If you are not sure, read the appropriate sections before making your selection.

[x] Life sciences [ ] Behavioural & social sciences [ ] Ecological, evolutionary & environmental sciences

For a reference copy of the document with all sections, see nature.com/documents/nr-reporting-summary-flat.pdf

Life sciences study design

All studies must disclose on these points even when the disclosure is negative.

Sample size	No sample size calculation was performed. n=3 biological triplicates were performed.
Data exclusions	No data was excluded from analysis.
Replication	All experiments were performed with three technical replicates and at least three independent biological replicates, sometimes more than five times. All attempts at replication were successful.
Randomization	In vitro experiments: cells were independently and randomly allocated into groups. For assays involving donor cells, each donor served as its own control (e.g. KD vs Control) before being subject to treatment (+/- stimulation).
Blinding	Blinding was not performed and is not relevant in this study.

Reporting for specific materials, systems and methods

We require information from authors about some types of materials, experimental systems and methods used in many studies. Here, indicate whether each material, system or method listed is relevant to your study. If you are not sure if a list item applies to your research, read the appropriate section before selecting a response.

Materials & experimental systems

n/a	Involved in the study
☐	☒ Antibodies
☐	☒ Eukaryotic cell lines
☒	☐ Palaeontology and archaeology
☒	☐ Animals and other organisms
☒	☐ Clinical data
☒	☐ Dual use research of concern

Methods

n/a	Involved in the study
☒	☐ ChIP-seq
☐	☒ Flow cytometry
☒	☐ MRI-based neuroimaging

Antibodies

Antibodies used

Mouse anti-HA (Zen-Bioscience, 201113) (1:1000); Rabbit anti-HA (Sigma-Aldrich, H6908) (1:3000) (3ug/test); Rabbit anti-H3 (Proteintech, 17168-1) (1:3000); Rabbit anti-Myc (Proteintech, 16286-1) (1:1000) (2ug/test); Goat anti-mouse IgG-HRP (Proteintech, SA00001-1) (1:3000); Goat anti-rabbit IgG-HRP (Proteintech, SA00001-2) (1:3000); Rabbit anti-TRIM5α (Cell Signaling Technology, 14326) (1:1000) (1ug/test); Rabbit anti-HDAC1 (Cell Signaling Technology, 34589) (1:1000) (1ug/test); Rabbit anti-H3K9ac (Cell Signaling Technology, 9649) (1ug/test); Rabbit IgG isotype control (Cell Signaling Technology, 2729) (1ug/test); Rabbit anti-HA (Cell Signaling Technology, 3724) (1:1000) (1ug/test); Mouse anti-IκBα (Cell Signaling Technology, 4814) (1:1000); Mouse anti-phospho-IκBα (Cell Signaling Technology, 2859) (1:1000); Rabbit anti-NF-κB p65 (Cell Signaling Technology, 8242) (1:1000); Rabbit anti-Sp1 (Cell Signaling Technology, 9389) (1:1000); Mouse anti-Flag (Cell Signaling Technology, 14793) (1:1000); Rabbit anti-LaminB1 antibody (Beyotime Biotec, AF5222) (1:1000); Anti-mouse Alexa Fluor 488 (Thermo Fisher Scientific, A21202) (1:2000); Anti-rabbit Alexa Fluor 555 (Thermo Fisher Scientific, A31572) (1:2000); Mouse IgG1 kappa isotype control (Thermo Fisher Scientific, 14-4714-85) (1:2000).

Validation

Mouse anti-HA (Zen-Bioscience, 201113) http://www.zenbio.cn/prod_view.aspx?IsActiveTarget=True&TypeId=189&Id=561049&FId=t3:189:3 and validated by correct kDa and over expression system.

Rabbit anti-HA (Sigma-Aldrich, H6908) https://www.sigmaaldrich.cn/CN/zh/product/sigma/h6908 and validated by correct kDa and over expression system.

Mouse anti-GAPDH (Proteintech, 60004-1) https://www.ptglab.co.jp/products/GAPDH-Antibody-60004-1-Ig.htm and validated by correct kDa.

Rabbit anti-H3 (Proteintech, 17168-1) https://www.ptglab.co.jp/Products/Histone-H3-Antibody-17168-1-AP.htm and validated by correct kDa.

Rabbit anti-Myc (Proteintech, 16286-1) https://www.ptglab.co.jp/Products/MYC-tag-Antibody-16286-1-AP.htm and validated by correct kDa and over expression system.

Goat anti-mouse IgG-HRP (Proteintech, SA00001-1) https://www.ptglab.co.jp/products/HRP-conjugated-Affinipure-Goat-Anti-Mouse-IgG-H-L-secondary-antibody.htm

Goat anti-rabbit IgG-HRP (Proteintech, SA00001-2) https://www.ptglab.co.jp/products/HRP-conjugated-Affinipure-Goat-Anti-Rabbit-IgG-H-L-secondary-antibody.htm

ChIP grade rabbit anti-TRIM5α (Cell Signaling Technology, 14326) https://www.cellsignal.cn/products/primary-antibodies/trim5a-d6z8l-rabbit-mab/14326 and validated by correct kDa, Knock-down and Knock-out assays.

Rabbit anti-HDAC1 (Cell Signaling Technology, 34589) https://www.cellsignal.cn/products/primary-antibodies/hdac1-d5c6u-xp-rabbit-mab/34589?site-search-type=Products&N=4294956287&Ntt=34589&fromPage=plp&_requestid=2117355 and validated by correct kDa.

Rabbit anti-H3K9ac (Cell Signaling Technology, 9649) https://www.cellsignal.cn/products/primary-antibodies/acetyl-histone-h3-lys9-c5b11-rabbit-mab/9649 and validated by correct kDa.

Rabbit IgG isotype control (Cell Signaling Technology, 2729) https://www.cellsignal.cn/products/primary-antibodies/normal-rabbit-igg/2729?site-search-type=Products&N=4294956287&Ntt=2729&fromPage=plp&_requestid=2117317

Rabbit anti-HA (Cell Signaling Technology, 3724) https://www.cellsignal.cn/products/primary-antibodies/ha-tag-c29f4-rabbit-mab/3724?site-search-type=Products&N=4294956287&Ntt=3724&fromPage=plp&_requestid=2117279 and validated by correct kDa and over expression system.

Anti-IκBα (Cell Signaling Technology, 4814) https://www.cellsignal.cn/products/primary-antibodies/ikba-l35a5-mouse-mab-amino-terminal-antigen/4814 and validated by correct kDa.

Anti-phospho-IκBα (Cell Signaling Technology, 2859) https://www.cellsignal.cn/products/primary-antibodies/phospho-ikba-ser32-14d4-rabbit-mab/2859?site-search-type=Products&N=4294956287&Ntt=2859&fromPage=plp&_requestid=2117402 and validated by correct kDa.

Anti-NF-κB p65 (Cell Signaling Technology, 8242) https://www.cellsignal.cn/products/primary-antibodies/nf-kb-p65-d14e12-xp-rabbit-mab/8242?site-search-type=Products&N=4294956287&Ntt=8242&fromPage=plp&_requestid=2117530 and validated by correct kDa.

Rabbit anti-Sp1 (Cell Signaling Technology, 9389) https://www.cellsignal.cn/products/primary-antibodies/sp1-d4c3-rabbit-mab/9389?site-search-type=Products&N=4294956287&Ntt=9389&fromPage=plp&_requestid=2117618 and validated by correct kDa.

Mouse anti-Flag (Cell Signaling Technology, 14793) https://www.cellsignal.cn/products/primary-antibodies/dykddddk-tag-d6w5b-rabbit-mab-binds-to-same-epitope-as-sigma-s-anti-flag-m2-antibody/14793?site-search-type=Products&N=4294956287&Ntt=14793&fromPage=plp&_requestid=2117670 and validated by correct kDa and over expression system.

Rabbit anti-LaminB1 antibody (Beyotime Biotec, AF5222) https://beyotime.com/product/AF5222.htm and validated by correct kDa.

Anti-mouse Alexa Fluor 488 (Thermo Fisher Scientific, A21202) https://www.thermofisher.cn/cn/zh/antibody/product/Donkey-anti-Mouse-IgG-H-L-Highly-Cross-Adsorbed-Secondary-Antibody-Polyclonal/A-21202 and validated by celluar localization.

Anti-rabbit Alexa Fluor 555 (Thermo Fisher Scientific, A31572) https://www.thermofisher.cn/cn/zh/antibody/product/Donkey-anti-Rabbit-IgG-H-L-Highly-Cross-Adsorbed-Secondary-Antibody-Polyclonal/A-31572 and validated by celluar localization.

Mouse IgG1 kappa isotype control (Thermo Fisher Scientific, 14-4714-85) https://www.thermofisher.cn/cn/zh/antibody/product/Mouse-IgG1-kappa-clone-P3-6-2-8-1-Isotype-Control/14-4714-85

Eukaryotic cell lines

Policy information about cell lines and Sex and Gender in Research

Cell line source(s)	293T cells were obtained from ATCC. TZM-bl cells and J-Lat cells were obtained from NIH AIDS Reagent program.
Authentication	All cell lines have not been authenticated.
Mycoplasma contamination	All cell lines were tested mycoplasma-free periodically by a PCR-based mycoplasma detection kit.
Commonly misidentified lines (See ICLAC register)	None of the commonly misidentified cell lines were used in this study.

Flow Cytometry

Plots

Confirm that:

- [x] The axis labels state the marker and fluorochrome used (e.g. CD4-FITC).
- [x] The axis scales are clearly visible. Include numbers along axes only for bottom left plot of group (a 'group' is an analysis of identical markers).
- [x] All plots are contour plots with outliers or pseudocolor plots.
- [x] A numerical value for number of cells or percentage (with statistics) is provided.

Methodology

Sample preparation	Cells were fixed with 4% paraformaldehyde prior to data acquisition.
Instrument	BD FACSverse cytometer.
Software	FACSuite 1.0.3 (BD); FlowJo 7.6.1 (TreeStar).
Cell population abundance	Each population contains at least 20,000 individual cells.
Gating strategy	Live population was gated out in the FSC-A and SSC-A; GFP positive population was gated out according to the unstimulated cell controls.

- [x] Tick this box to confirm that a figure exemplifying the gating strategy is provided in the Supplementary Information.
